# Seeing Through Muddy Water: Laser‐Induced Graphene for Portable Tomography Imaging

**DOI:** 10.1002/advs.202406905

**Published:** 2024-07-15

**Authors:** Haosong Zhong, Xupeng Lu, Rongliang Yang, Yexin Pan, Jing Lin, Minseong Kim, Siyu Chen, Mitch Guijun Li

**Affiliations:** ^1^ Center on Smart Manufacturing Division of Integrative Systems and Design The Hong Kong University of Science and Technology Clear Water Bay, Kowloon Hong Kong SAR China

**Keywords:** electrical impedance tomography, flexible electronics, laser‐induced graphene, resistance sensing, wearable devices

## Abstract

Due to its outstanding physical and chemical properties, graphene synthesized by laser scribing on polyimide (PI) offers excellent opportunities for photothermal applications, antiviral and antibacterial surfaces, and electrochemical storage and sensing. However, the utilization of such graphene for imaging is yet to be explored. Herein, using chemically durable and electrically conductive laser‐induced graphene (LIG) for tomography imaging in aqueous suspensions is proposed. These graphene electrodes are designed as impedance imaging units for four‐terminal electrical measurements. Using the real‐time portable imaging prototypes, the conductive and dielectric objects can be seen in clear and muddy water with equivalent impedance modeling. This low‐cost graphene tomography measurement system offers significant advantages over traditional visual cameras, in which the suspended muddy particles hinder the imaging resolution. This research shows the potential of applying graphene nanomaterials in emerging marine technologies, such as underwater robotics and automatic fisheries.

## Graphical abstract

Electrical Tomography imaging with laser‐induced graphene electrodes as portable electrode arrays to see through nonvisible objects in muddy water. The laser‐induced graphene enables the high accuracy of the spatial distribution of electrodes and high durability in a salty solution, with high potential to be used in salty aqueous conditions, such as undersea robotics imaging applications.

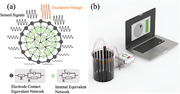

## 1 Introduction

Electrical Tomography (ET) is an attractive non‐invasive imaging technology that visualizes the internal conductivity or permittivity distribution within a two‐dimensional (2D) or three‐dimensional (3D) region of interest through electrical signal measurements on the boundary. A typical ET system consists of an electrode array affixed to the external surface of the region, a multiplexing measurement device, and a computer for image reconstruction. As the invisible conductivity/permittivity distribution changes, there is distinct attenuation on electrical signals injected from different electrode pairs, enabling the inverse estimation of a heat map depicting the changes. Resulting from the merits of non‐invasion, cost‐effectiveness, fast response, and environmental sustainability, ET has been widely applied in various fields, including healthcare,^[^ ^1^ ^]^ industrial process control,^[^ ^2^ ^]^ and environmental monitoring.^[^ ^3^ ^]^

In recent years, various imaging technologies, such as ultrasonic, radiation, and magnetic imaging, have been developed to satisfy applications where charge‐coupled device (CCD) cameras fail to provide accurate imaging. Due to varying levels of attenuation and reflection for acoustic signals, ultrasonic imaging reconstructs medium‐distribution images from reflected waves based on digital signal processing technology. Li et al. proposed a method to compensate for transducer component delays, leading to improvements in underwater imaging.^[^ ^4^ ^]^ The technology can provide clear, velocity‐sensed, and real‐time images with a budget for complex transducer elements and worse responses on multi‐phase interfaces. Like ultrasonic imaging, radiation imaging utilizes the projected X–/γ–rays to obtain the attenuation difference of invisible substances. Riis et al. successfully applied radiational tomographic reconstruction on underwater pipeline inspection.^[^ ^5^ ^]^ The technology can stably reconstruct high‐resolution distribution images, however, it has the disadvantages of high costs and ionizing radiation emissions. Magnetic imaging also finds extensive applications in underwater inspection,^[^ ^6^ ^]^ atherosclerosis diagnosis,^[^ ^7^ ^]^ and mineral research.^[^ ^8^ ^]^ Yuan et al. developed a magnetic flexible sensor array for nondestructive testing of underwater cracks, thus achieving low average length, depth, and angle errors.^[^ ^6^ ^]^ The technology has the advantage of insensitivity to the fluctuation of the measured objects, whereas the magnetic signals are easily interfered by external noises.

Considering the drawbacks of large equipment volume, high costs, and environmental pollution associated with the mentioned technologies, Electrical Impedance Tomography (EIT), a segmented field from ET, provides a potential alternative. The functionality of an EIT device can be implemented in an intelligent watch‐sized wearable device. With the capability of wireless communication protocols such as Bluetooth and Wi‐Fi, such a wearable EIT device can implement long‐term, in‐situ health telemonitoring,^[^ ^9^ ^]^ eliminating concerns regarding toxicity from heavy metals in metallic electrodes.^[^ ^10^ ^]^ This has been verified in lung respiratory imaging and intracerebral hemorrhage imaging. Recently, attractive advancements have been made in EIT, particularly in hardware, reconstruction algorithms, and electrode materials. Bai et al. developed a modified noise model that obviously simplifies the optimization process of the EIT system.^[^ ^11^ ^]^ Liu et al. proposed an algorithm based on sparse Bayesian learning, enhancing the image resolution and parameter tuning process through the structure‐aware priors.^[^ ^12^ ^]^ The D‐bar method and deep learning methods have also been taken into account.^[^ ^13^ ^]^ Metals like copper are widely used as electrodes because of their malleability and low resistance. For example, one major experiment for EIT is reconstructing the cross‐sectional permittivity distribution map inside a water tank. However, metals react with electrolyte water, resulting in serious metallic corrosion. The process is significantly accelerated by the injected current and electrochemical potential, generating thin oxide, thick chloride, or even an oxychloride layer.^[^ ^14^, ^15^ ^]^ Thus, several researchers explored EIT systems with non‐metal electrodes such as AgCl bioelectrodes,^[^ ^16^ ^]^ dry textile electrodes,^[^ ^17^ ^]^ electrode gel,^[^ ^18^ ^]^ conductive hydrogel,^[^ ^19^ ^]^ commercial electroencephalography electrodes,^[^ ^20^ ^]^ and nanofiber web.^[^ ^21^ ^]^ However, adhesive electrodes could be uncomfortable and raise the possibility of skin damage during extended measurement periods.^[^ ^17^ ^]^ Furthermore, a number of adhesive electrodes are harmful to environments containing large amounts of particle pollution, like solar evaporation experiments and underwater robotics. According to previous research, EIT devices working in four‐terminal mode are insensitive to contact resistances,^[^ ^22^ ^]^ providing greater flexibility in selecting materials for EIT electrodes.

Laser‐induced graphene (LIG) is a high‐conductivity carbon nanomaterial synthesized by laser‐induced carbonization of certain polymer materials (e.g., polyimide).^[^ ^23^ ^]^ As compared to other carbon nanomaterials, including graphene nanosheets or carbon nanotubes, LIG can form continuous patterns without extra assembly processes. This characteristic is ideally suitable for wearable electronics, especially EIT electrodes. The laser writing process can partially carbonize the aromatic‐hydrocarbon‐based polymer into graphitized carbon, thus forming customized LIG patterns on the polymer film. Since the serendipitous discovery of LIG was reported,^[^ ^24^ ^]^ customized LIG patterns have been used as flexible non‐metallic circuitry for various applications. Using LIG's property of good electrical conductivity, Yang et al. proposed a LIG sensor and a flexible monitoring device for continuous sweat detection and analysis.^[^ ^25^ ^]^ A triboelectric sensor array has been developed based on patterned LIG materials for tracking the fingers and flexible screen application.^[^ ^26^ ^]^ Zhang et al. designed LIG‐based interdigitated electrodes for micro‐supercapacitors, achieving a fantastic electrochemical performance.^[^ ^27^ ^]^ Patterned LIG materials also have demonstrated the feasibility of Li‐battery application and have been proven as the well‐performed nucleation position for Li‐ion deposition.^[^ ^28^ ^]^ Huang et al. developed a urea electrochemical sensor capable of telemonitoring based on LIG circuits, thus demonstrating LIG's superior chemical resistivity.^[^ ^29^, ^30^ ^]^ Compared to metallic circuitries, LIG‐based circuitries show higher flexibility and resistance to corrosion. As a result, they are better suited for those applications that operate in humid or aqueous environments.

This work develops a corrosion‐resistant EIT system composed of innovative LIG electrodes and a specifically designed EIT device for long‐term underwater monitoring. The application of LIG electrodes contributes to a reduction in cost and chemical stability, thus enabling the widespread adoption of EIT with efficient manufacturing processes. The chemical stability of LIG electrodes also benefits four‐terminal impedance sensing, allowing the circuit to mitigate errors arising from direct‐current (DC) bias voltage and the unstable resistivity of LIG. In this study, LIG electrodes were fabricated on a flexible substrate using direct laser writing with a 1064 nm laser, and their evaluation was conducted in an EIT experimental setup. Such a fabrication eliminates the requirement for an external high‐temperature thermal source and toxic chemicals.^[^ ^31^ ^]^ Furthermore, we also developed three versions of the EIT data acquisition circuit. All these circuit prototypes were assembled and tested with the fabricated flexible LIG electrode array to build the experimental setup. Our work proved that LIG fabrication on the PI substrate is an effective method for manufacturing EIT electrode arrays. They perform well under various test cases and offer a similar imaging quality to metal electrodes with temporary anti‐corrosion features.

## 2 Results and Discussion

**Figure 1:**
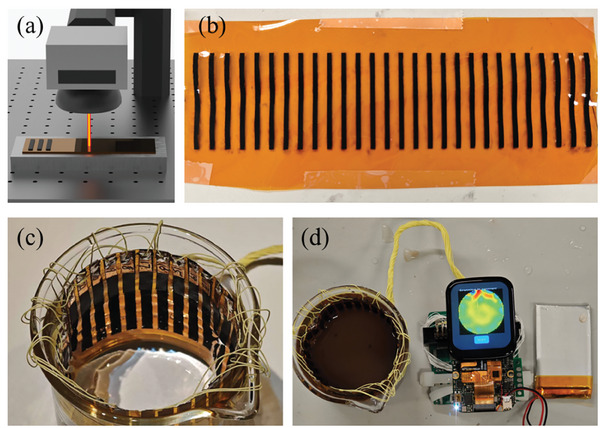
a) The fabrication of LIG electrodes using a fiber laser marking machine. b) The fabricated LIG electrodes with 1064 nm fiber laser. c) Testing LIG electrodes inside a beaker with water for EIT application. d) A prototyped EIT testing experimental setup with LIG electrodes used.

**Figure** **1** illustrates the fabrication process of a flexible LIG electrode array and the complete experimental setup. Figure 1a illustrates the schematic of LIG fabrication in which a laser marking machine (fiber laser with a wavelength of 1064 nm) is used. Additionally, an external heat sink is used to assist with excessive heat dissipation. The fabricated flexible electrodes, as indicated in Figure 1b, are well aligned with a black color. Such electrodes are bent into a cylinder shape inside a beaker, as depicted in Figure 1c, and connected to the EIT readout and inverse‐solving device, as shown in Figure 1d.

**Figure 2:**
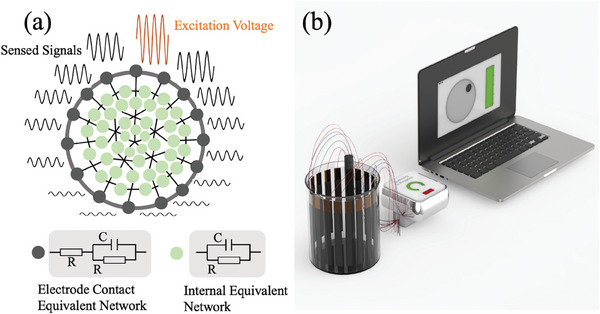
a) The simplified equivalent impedance modeling of the sample. b) The setup of the EIT experiment: The EIT data acquisition device measures the potential difference distribution across different electrodes under different excitation protocols. It sends these measurements via Bluetooth Low Energy to a computer using the EIT inverse solver algorithm.

The EIT device provides an excitation voltage and successfully senses the potentials across the 6 sensory electrodes every 2 sine wave periods. It computes the root mean square(RMS) voltage for each electrode pair, as shown in **Figure** **2**a, which is repeated 32 times to complete a cycle of EIT data acquisition. Such a result indicates a voltage pattern shaped in the form of a U‐curve, as can be seen in Figure S1a,c (Supporting Information), while the LIG electrode sample demonstrates greater DC‐offsets than the copper electrode sample, as shown in Figure S1b,d (Supporting Information). These 928 RMS voltages are used to compute a frame of conductivity mapping on the device or an external computer, as shown in Figure 2b.

**Figure 3:**
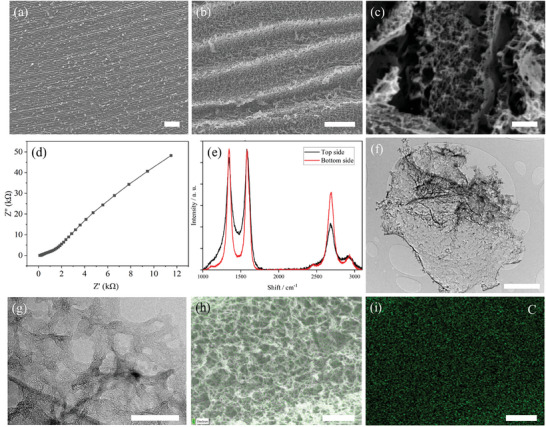
a–c) SEM image of the EIT LIG electrode sample; the scale bars are 100, 10, and 2 µm, respectively. d) EIS testing result of the EIT LIG electrode. e) Raman characterization of the EIT LIG electrode. f,g) TEM images of the EIT LIG electrode sample; the scale bars are 1 and 100 nm, respectively. h,i) EDX mapping result, the scale bars are 10 µm.

The EIT electrode scanning electron microscopy (SEM) images (**Figure** **3**a–c) are captured using a JEOL‐7100F field emission scanning electron microscope. SEM images show that the sample (Figure 3a,b) has a porous structure. The sample is mechanically bendable and not brittle. It attaches tightly to the polyimide substrate, which is suitable for underwater robotics applications that require high reliability.^[^ ^32^ ^]^ In Figure 3c,d, the porous structure proves the formation of LIG on the PI substrate.^[^ ^33^, ^34^ ^]^ The electrical impedance spectroscopy (EIS) plot (Figure 3f) shows the charge transfer and resistance characteristics of the fabricated LIG electrode. The transmission electron microscopy (TEM) images of LIG (Figure 3f,g) display the micro‐sized nanoflakes with mesopores and macropores, which could create ion‐storage regions within the electrode, thus causing a reduction in the electrolyte ion diffusion path. In addition, a Raman spectrum showing a D, G, 2D, and D+G peak also indicates that carbonization occurred and LIG was formed.^[^ ^35^ ^]^ The results of energy dispersive X‐ray spectrum (EDX) mapping and elemental analysis (Figure 3h,i) indicate that the fabricated LIG mainly comprises evenly distributed carbon. By controlling the laser parameters, super‐hydrophilic characteristics can be obtained on the LIG electrodes for a reduced electrode‐liquid interface impedance to improve signal quality.

**Figure 4:**
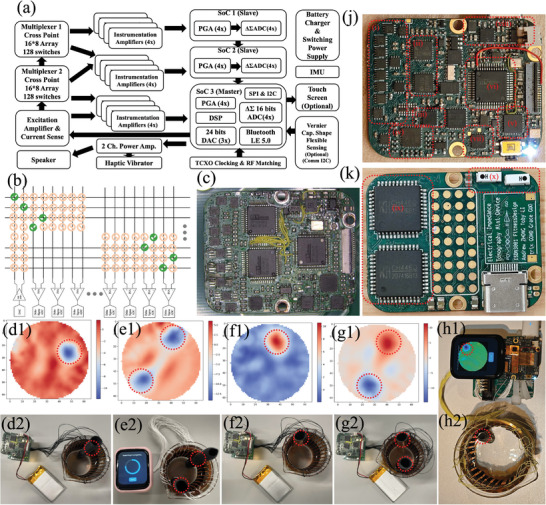
a) Block diagram of the portable EIT data acquisition device. b) Electrical connections between multiplexers and instrumentation amplifiers simplify EIT data acquisition. c) The EIT data acquisition device. d2, e2, f2, g2) EIT testing setup and its respective reconstructed conductivity distribution map d1, e1, f1, g1). The red dashed circle indicates the items and the corresponding indication on the reconstructed image. h) The whole EIT system for testing. j) The front side of the EIT device, including i) Instrumentation amplifiers, ii) Delta‐sigma ADC converters, iii) Operational amplifier, iv) Resistor multiplexer, v) Realtek WiFi communication chip, vi) BP1048P4 Bluetooth SoC, vii) Class‐D amplifier, viii) Switching mode power supplies. k) The back side of the EIT device, including ix) Matrix switches, x) Bluetooth, and Wi‐Fi ceramic antennas.

We designed an EIT data acquisition device (Figure S2b–g, Supporting Information) with the block diagram shown in **Figure** **4**a, the electrical schematic diagram in Figure S3 (Supporting Information), the circuit board diagram in Figure S4 (Supporting Information), and multiplexer operation schemes in Figure 4b. The final version of the EIT device supports Bluetooth communication and mobile application. Figure S5a (Supporting Information) shows the 5.51266 kHz sine wave excitation signal. Figure S5b,c (Supporting Information) shows the waveform measured on the EIT electrode. The developed system prototype yields all the results shown in Figure 4. Figure 4d,e shows the actual positions of various objects and the reconstructed conductivity distribution map with the Tikhonov algorithm running on an embedded microcontroller, respectively.^[^ ^36^ ^]^ Such testing indicated that the LIG electrodes and the whole setup function normally, as expected in a clear water situation.

**Figure 5:**
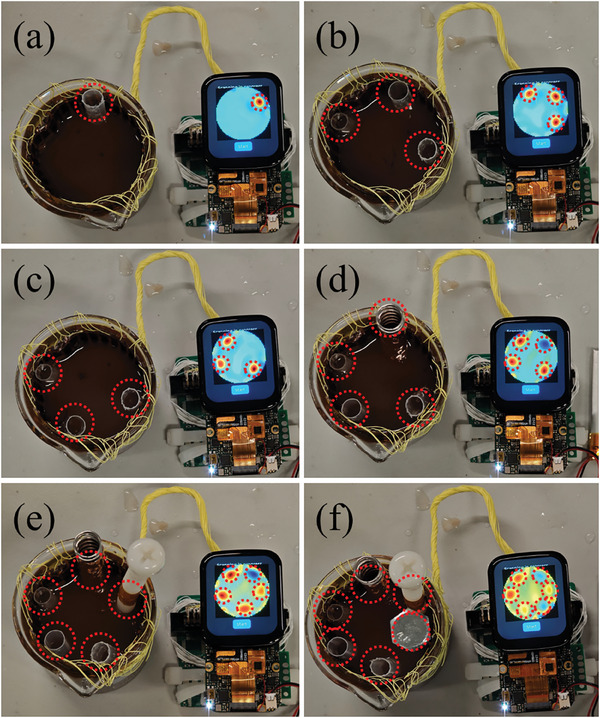
a) EIT testing setup with LIG electrodes, muddy water, and on‐device inverse solving algorithm with one low‐conductivity object. b,c) Three low‐conductivity objects with different positions. d) Three low‐conductivity objects and one high‐conductivity object. e) One high‐conductivity object and four low‐conductivity objects. f) Two high‐conductivity objects and four low‐conductivity objects. The red dashed circle indicates the items and the corresponding indication on the reconstructed image.

**Figure** **5** shows the ground truths with different numbers of high‐conductivity or low‐conductivity items. Each conductivity distribution map is reconstructed with the Tikhonov algorithm (alpha = 0.2),^[^ ^1^ ^]^ displayed on the liquid crystal display (LCD) screen respectively. The whole process runs on Bluetooth SoC with a Fourier transform accelerator and digital signal processing (DSP) library.^[^ ^14^ ^]^

To illustrate the advancements of LIG electrodes, an electrochemical corrosion experiment is conducted to verify the anti‐corrosion performance compared with metal electrodes (Figure S6, Supporting Information). In the experiment, two kinds of electrodes fabricated with copper and LIG were studied (Figure S7, Supporting Information). After soaking in seawater and being injected with a 3‐volt electrical signal for 30 minutes, apparent copper rust can be observed on the copper electrodes (Figure S7b, Supporting Information), and an evident blue color can be observed in the solution (Figure S7a, Supporting Information). In contrast, Figure S7b (Supporting Information) illustrates that no visible change is observed on the LIG electrodes, indicating long‐term stability.

These findings provide a new solution that fills the existing research gap. It equips the EIT industry with a cost‐effective, easily fabricated electrode array for corrosive applications such as underwater robotics and seawater solar evaporation. The EIT LIG electrodes exclude corrosion‐yielded toxicity, which affects the health of marine life. In addition, a flutter‐based application has been developed to study the time‐elapsed characteristics of EIT data (Figure S8, Supporting Information).

Although researchers have proven that multiple materials can be used as EIT electrodes, some do not last long enough to maintain a stable electrical connection. Sweat and grease are usually contaminations found on human skin,^[^ ^37^ ^]^ which react with EIT electrode material and cause metallic corrosion because of the presence of salt, lactic acid, and urea.^[^ ^38^ ^]^ All the EIT devices inject an excitation current into the measured sample, which produces a voltage between the conductive sample and the electrode. The electrochemical potential forms a thin oxide layer, a thick chloride layer, or even an oxychloride layer.^[^ ^38^ ^]^ A higher connection count in EIT electrodes yields a more precise imaging performance in spatial resolution. However, a higher connection count of the EIT electrode creates fabrication difficulties in circuit patterning. The tiny and thin metallic electrodes can be eroded quickly in a seawater evaporation experiment setup containing EIT electrodes. Laser interaction with materials has been extensively studied recently due to the emergence of solid‐state laser technologies, fast‐switching controlled lasers, and breakthroughs in the power density of devices.^[^ ^39^ ^]^ LIG is a 3D, multilayer graphene fabricated at high temperatures in a tiny area generated by the laser, which causes the carbon atoms in the substrate to undergo a carbonization process.^[^ ^23^ ^]^ The carbon precursors are typically polymer‐based materials such as polyimide. This novel method of fabricating EIT electrode arrays provides a low‐cost, easy, and stable solution for applications like seawater evaporation. As opposed to other patterning methods of fabricating functional materials, the fabrication of LIG does not involve using solvents, reducing the environmental budget.^[^ ^40^ ^]^ The speedy responses of laser galvos and modern controlling systems enable a high‐throughput fabrication with scalability.^[^ ^40^ ^]^ Unlike metallic electrodes such as copper, no heavy metal is involved in fabricating our LIG electrodes. As marine applications need EIT electrodes installed underwater, metals might be corroded by seawater and release harmful substances to the water body. Although anodic protection (AP) or cathodic protection (CP) can protect the electrode, such a method does not stop corrosion from happening, which might still have potentially harmful effects on the environment and cause cost‐effectiveness issues.

This is the first work proving that LIG fabricated by a 1064 nm fiber laser can be used as EIT electrode material. Figure 5f shows that an EIT system based on our novel LIG electrodes can distinguish up to 6 objects in an experimental setup. The superior precision of laser fabrication ensures that the electrode distances ratios in the finite element modeling (FEM) model match the exact experimental setup well. The obtained result demonstrates that the EIT device can effectively compensate for the additional resistances introduced by LIG material. It benefits from four‐terminal EIT mode and multiple channels of 16‐bit resolution simultaneous sensing. This compensation allows the LIG electrodes to deliver performance comparable to that of metallic electrodes.

This research has limitations on the wettability study of LIG electrodes against other liquids. When conducting EIT measurements in an environment with a high presence of lipids, utilizing hydrophilic LIG electrodes reduces contact resistance. It establishes a strong connection between the sample and EIT electrodes, resulting in an improved signal‐to‐noise ratio (SNR).^[^ ^41^ ^]^ Controlling the laser parameters for a dedicated wetting status of electrode arrays is a cost‐effective fabrication method.^[^ ^42^ ^]^ The material between two EIT electrodes could be covered by a superhydrophobic layer of LIG, which resists the water absorption on some materials (such as fabrics) for a reduced crosstalk between different electrodes. The precision of the entire EIT measurement apparatus has not been adequately shown due to the limited rate of analog‐to‐digital converter (ADC) sampling speed and bits of ADC resolution in the embedded processor of the data acquisition device. The switching noise from multiplexers, Bluetooth radio frequency interference, gain mismatch between multiple delta‐sigma ADCs and instrumentation amplifiers, temperature drift in the internal voltage reference of ADC, and uneven spacings between each electrode contribute to the overall systematic error. Besides, although the Tikhonov algorithm does not have the highest accuracy compared to other algorithms, its non‐iterative computation and low memory footprint feature enable real‐time image reconstruction in the resource‐limited embedded device.^[^ ^43^ ^]^ The size, shape of each electrode, and spacings between each electrode are not the same as the sensitivity matrix calculated by FEM used in the Tikhonov inverse solver,^[^ ^44^ ^]^ leading to minor errors in EIT image reconstruction. Due to the speed limitation of this experiment's EIT data acquisition unit, the speed benefit of replacing metal EIT electrodes with LIG electrodes for reduced inter‐electrode DC offsets is not evaluated.

Improving versatility, flexibility, contact stability, and cost reduction in mass production are potential future directions of this work.^[^ ^45^ ^]^ Enhancing SNR, sampling speed, rejection of unstable contact resistances, and form‐factor optimizations are potential areas where future improvements could be made to the EIT data acquisition device. As EIT LIG electrodes have much higher resistivity than metals, the investigation of signal quality affected by the shape and connection of LIG electrodes needs to be conducted. Improving the four‐terminal EIT device's electrode contact resistance rejection is also crucial for LIG electrode EIT devices.^[^ ^46^ ^]^ Additionally, further experiments can be conducted using LIG material for the electrodes and the flexible circuit connections between the EIT acquisition unit and electrode arrays to reduce cost. Exposing zebrafish to low doses of LIG has not exhibited clear toxic responses. Therefore, it is imperative to conduct future experimental investigations by application of LIG electrodes for impedance imaging on biological entities. These experiments aim to explore the variances in contact impedance between LIG and metals across the skin‐to‐electrode interface.^[^ ^37^, ^47^ ^]^ In the future, installing our innovative LIG electrode array as a fixed setup in a marine environment can verify the in‐situ and dynamic real‐time imaging performance. Corresponding finite element models can be set up according to the actual positions of electrodes on the surface of underwater robotic devices such as underwater drones or automated fish robots to investigate the EIT performance on simulations of marine applications.^[^ ^32^ ^]^

## 3 Conclusion

Despite the optimization of different algorithms, readout circuits, and electrode materials for diverse applications, most EIT devices encounter problems with electrode degradation, leading to a decreasing signal quality during long‐term usage. Our work contributes to bridging the EIT study gap between theoretical, computational, and electronics research. EIT portable devices, reconstruction algorithms, and applications are connected in a stable, cost‐effective, and environmentally friendly way, especially for underwater robotics and automation. Our LIG material, fabricated via a commercial fiber laser machine, verifies its feasibility as the EIT electrodes in muddy water situations. Despite the higher resistance of LIG electrodes as compared to metals, it does not affect the performance of four‐terminal EIT systems due to the insensitivity to contact impedance within an acceptable range. Besides, our innovatively fabricated LIG electrodes demonstrate superior cost‐effectiveness, environmental protection, electrical stability, and mechanical performance. Simultaneously, our specifically designed device effectively accommodates a broad range of contact impedance levels and offers high performances of 16‐bit resolutions, multi‐channel simultaneous, continuous sampling, optimizing the resolution and frame rate when utilizing LIG electrodes. Such a complete set of EIT systems is available in a watch‐sized device, which can be seamlessly integrated into underwater robotics or marine automation devices.

## 4 Experimental Section

### Preparation of EIT LIG Electrode Arrays

The EIT electrode arrays in this work were fabricated on polyimide film with a 1064 nm fiber laser beam (Figure 1a). The PI film with a thickness of 150 µm was used directly after being purchased with no treatment.^[^ ^24^ ^]^ Then, it was taped on an aluminum alloy heat sink with transparent tape. De‐ionized (DI) water was filled in the small gap between the PI film and the heat sink. The pattern of 32 electrodes was drawn in EzCAD software, with laser scanning lines parallel to the short edge and a line distance of 0.03 mm. The PI absorbed laser power during the scanning, and the graphene flakes could be synthesized. The aluminum alloy below absorbed excessive heat conducted by water from the PI film to prevent puncturing caused by the over‐burning of this film. To avoid excessive curling of the PI film causing potential alteration of the focal distance, substantial force was applied to the transparent tape to ensure the adhesion to the aluminum heat sink and effectively tension the PI film. 32 electrodes were fabricated piece‐by‐piece in the PI film, and each piece of LIG electrode took 4 s to fabricate. A fiber laser scribing machine was used for the LIG fabrication with a 1064 nm laser, 32.5 cm focal length, and 350 mm s^−1^ scribing speed. Fabricating LIG with 1064 nm requires sticking the PI film on glass with tension applied by tape. No water was filled between the PI film and glass, and a 32.5 cm focal distance (matched with the F‐theta lens) was used. The pattern of 32 electrodes was drawn in Diaotu Industrial software, with laser scanning lines parallel to the short edge and a line distance of 0.013 mm. The PI absorbed laser power during the laser scanning, and the graphene flakes could be synthesized on a double side of the PI film.

After LIG was fabricated on the PI substrate, a sheet of shielding fabric tape with conductive adhesive was stuck on one side of the electrode array, with an overlapping area on the LIG. The conductive shielding tape was cut using a soldering iron with a temperature of 210 °C from 32 independent electrode connections. Polytetrafluoroethylene (PTFE)‐coated wires were soldered on conductive fabric tape with a temperature of 230 °C. The soldering alloy was SAC305 with 96.5% tin, 3% silver, and 0.5% copper. The laser process windows for fabricating adhesively attached LIG material on PI film were narrow, and the temperature change in the laser tube might lead to a significant change in parameters, hindering the LIG formation. Such a difficulty needs to be compensated for mass production and might be optimized with technical developments in the future. For a comparison experiment and verification of the correctness of the EIT device, an experimental setup with an array of copper electrodes was also prepared (Figure S6, Supporting Information). The copper electrodes were cut by scissors, each piece ≈ 13 mm × 130 mm, and stuck onto the inner side of a water bucket.

### Design of EIT Data Acquisition Device

The adopted EIT data read‐out unit was a four‐terminal EIT device. Two array switch multiplexers (CH446Q) from Nanjing QinHeng Electronics routed the electrical connections of each electrode to an excitation amplifier output or an instrumentation amplifier input. The instrumentation amplifier (INA350) from Texas Instruments with two selectable voltage gains (G = 10 or 20 V/V) amplified the voltage and currents of the signal from EIT sensing pairs routed from the multiplexers. The amplified signal entered the Delta‐Sigma ADC in the Bluetooth SoC (ShangHai MVSilicon BP1064L2). The four ADCs sampled three EIT sensing pairs and EIT excitation current, respectively. The digital‐to‐analog converter (DAC) generated variable frequency sine waveforms for EIT excitation. The single‐end signal output from the DAC was amplified by an operational amplifier from SGmicro (SGM3130) and to forma complementary output. The output current from one operational amplifier was sensed by an instrumentational amplifier (INA350), and feedback signal was provided to the Bluetooth SoC. The injected current's frequency was 5512.66 Hz, and the injected voltage was 5.8 Volts peak‐to‐peak (complementary output excitation) (Figure S5a, Supporting Information). The response signals were measured by Tektronix MDO3024 mixed domain oscilloscope. In each frame of EIT data acquisition, the excitation pair switched 32 times (Figure S5b, Supporting Information). Each 2 cycles of the signals on each sensing pair were sampled to calculate the alternating current (AC) RMS voltage value (Figure S5d, Supporting Information).

Because of the redox reaction, a DC voltage existed between the electrode and the measured object. Each electrode had a unique potential due to the varying amounts of substances participating in the response. The potential difference between different electrodes contributed to the DC offset voltage at the input of the instrumentation amplifier. This varying voltage would cause the saturation data acquisition in the analog‐to‐digital converter (ADC) if the gain was not set correctly. Even though automatic gain control (AGC) or offset calibrating can be employed, filtering out or compensating for a varying DC voltage leads to a smaller portion of the ADC range utilized for sampling the AC voltages. It reduces the overall systematicsignal‐to‐noise ration (SNR) in EIT voltage measurement.^[^ ^48^ ^]^

## Conflict of Interest

The authors declare no conflict of interest.

## Author Contributions

H.Z. and X.L. contributed equally to this work. M.G.L. conceived the idea and supervised the research. H.Z. and X.L. drafted the manuscript and worked on conceptualization and methodology. H.Z., X.L., R.Y., Y.P., and J.L. conducted the experiments. S.C. worked on characterization. M.K. worked on visualization. H.Z. worked on hardware and software development. All authors discussed, revised, and finalized the manuscript.

## Supporting information

Supporting Information

Supplemental Movie 1

## Data Availability

The data that support the findings of this study are available from the corresponding author upon reasonable request.
