# Supplementary material for: Seeing Through Muddy Water: Laser‐Induced Graphene for Portable Tomography Imaging
Source: Adv Sci (Weinh). 2024 Jul 15;11(35):2406905. doi: 10.1002/advs.202406905 (PMC11425229; doi:10.1002/advs.202406905)
Supplement: Supplementary file 1 — Supporting Information [file ADVS-11-2406905-s001.docx]

Supporting Information

Seeing through Muddy Water: Laser-induced Graphene for Portable Tomography Imaging

Haosong Zhong, Xupeng Lu, Rongliang Yang, Yexin Pan, Jing Lin, Minseong Kim, Siyu Chen, Mitch Guijun Li*


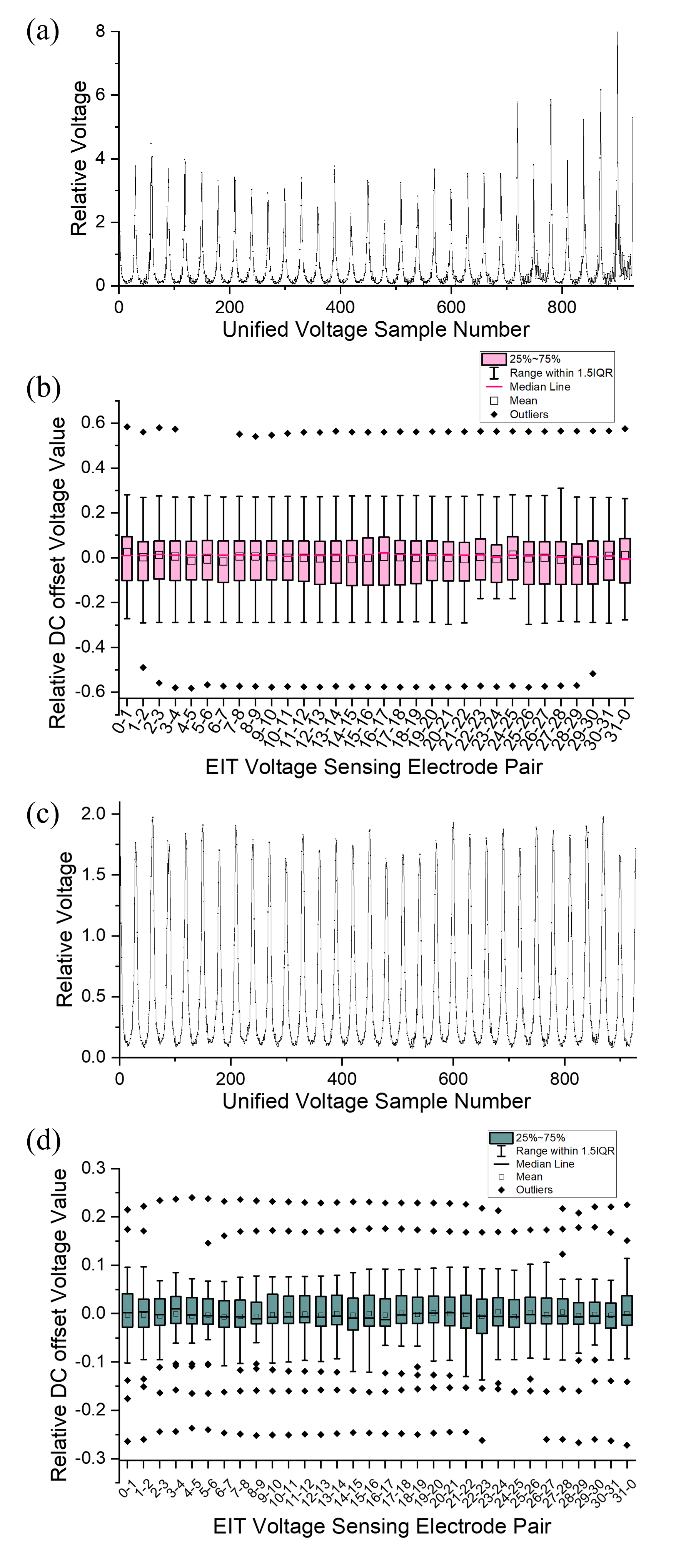


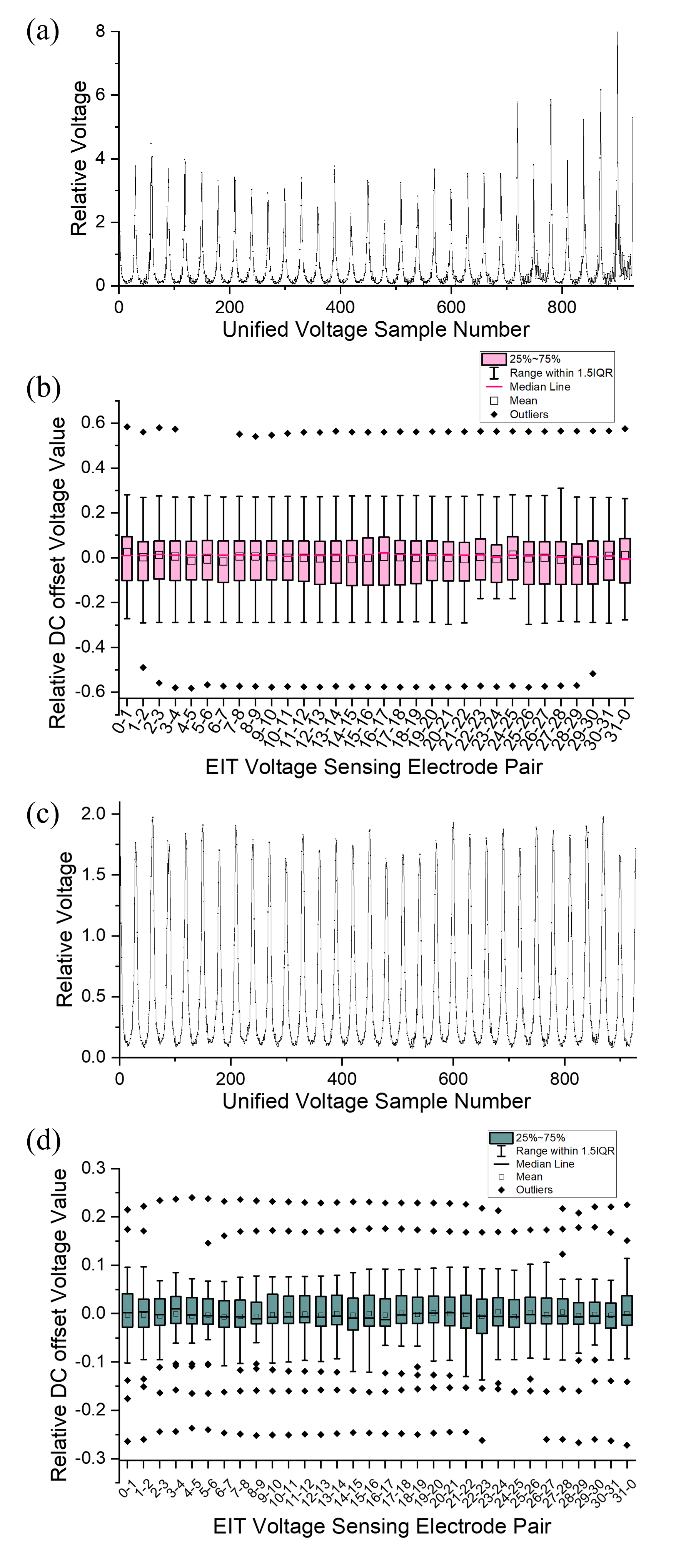


**Figure S1.** a) Relative Unified voltage samples from the device connected to the EIT testing setup with LIG electrodes. b) DC offset voltage distributions on each EIT sensing LIG electrode pair. c) Relative Unified voltage samples from the device connected to the EIT testing setup with copper electrodes. d) DC offset voltage distributions on each EIT sensing copper electrode pair.


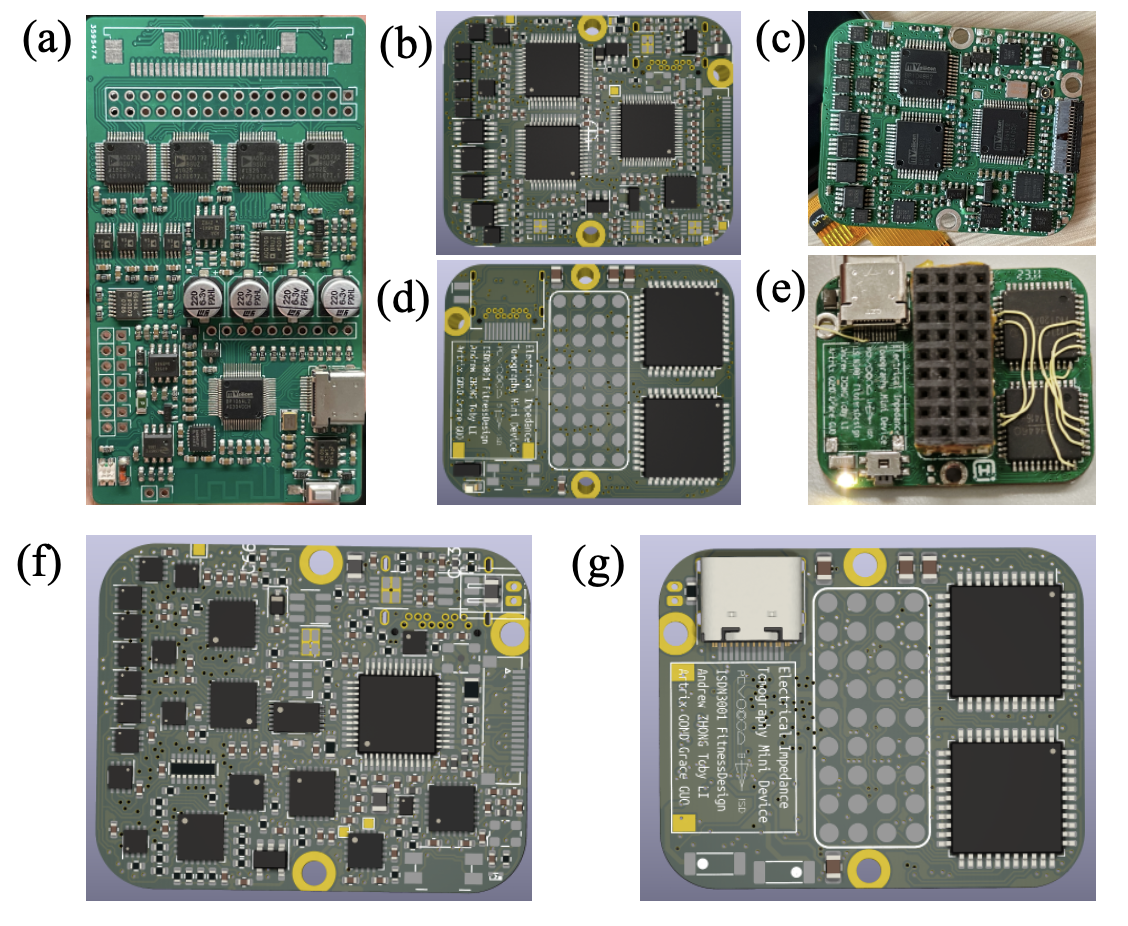


**Figure S2.** a) The specially designed EIT device (The first version). b, d) Rendered 3D diagram of the front and back sides of the EIT device (the second version). c, e) The front and back sides of the EIT device (the second version). f, g) Rendered 3D diagram of the front and back sides of the EIT device (the third version).


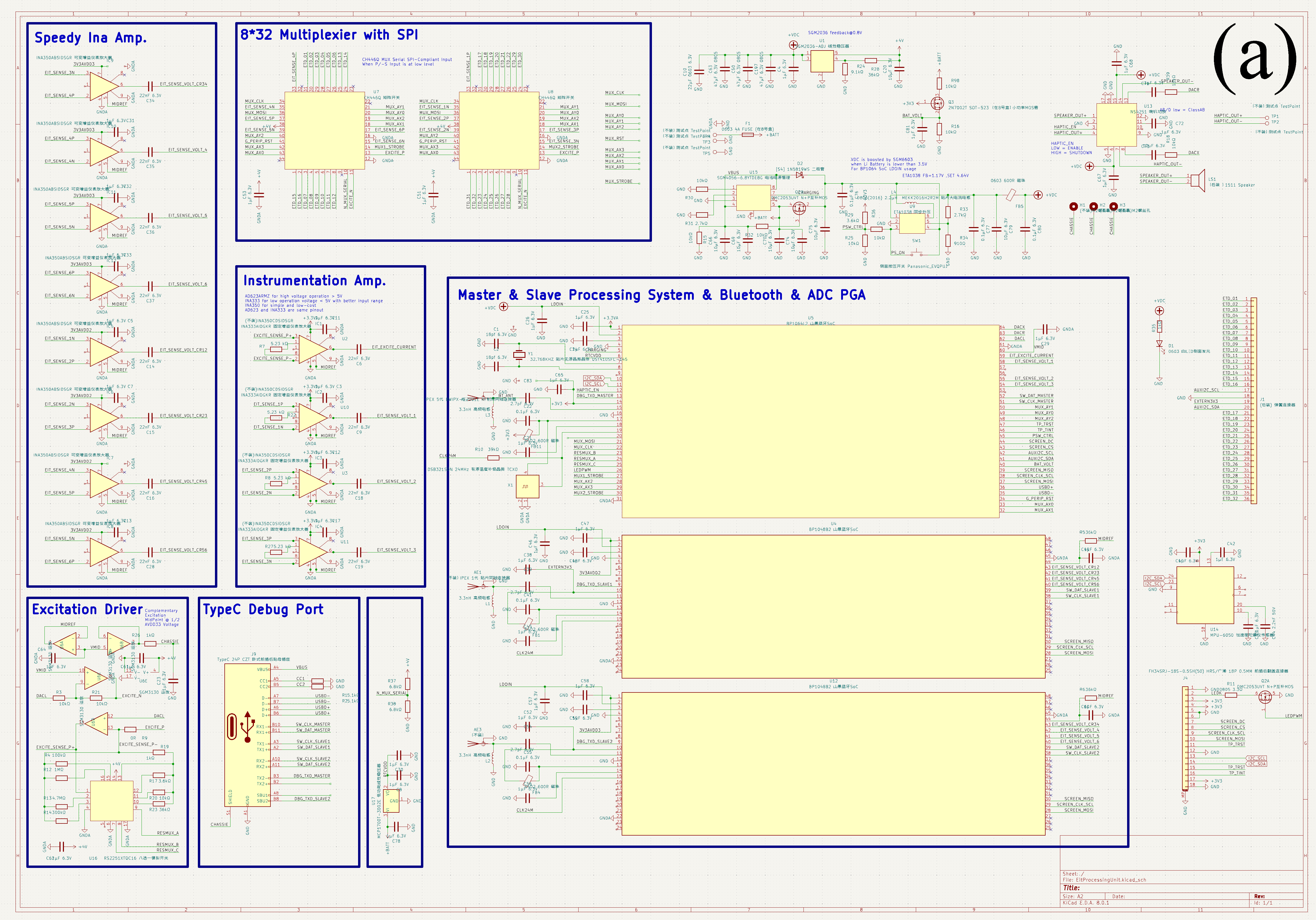


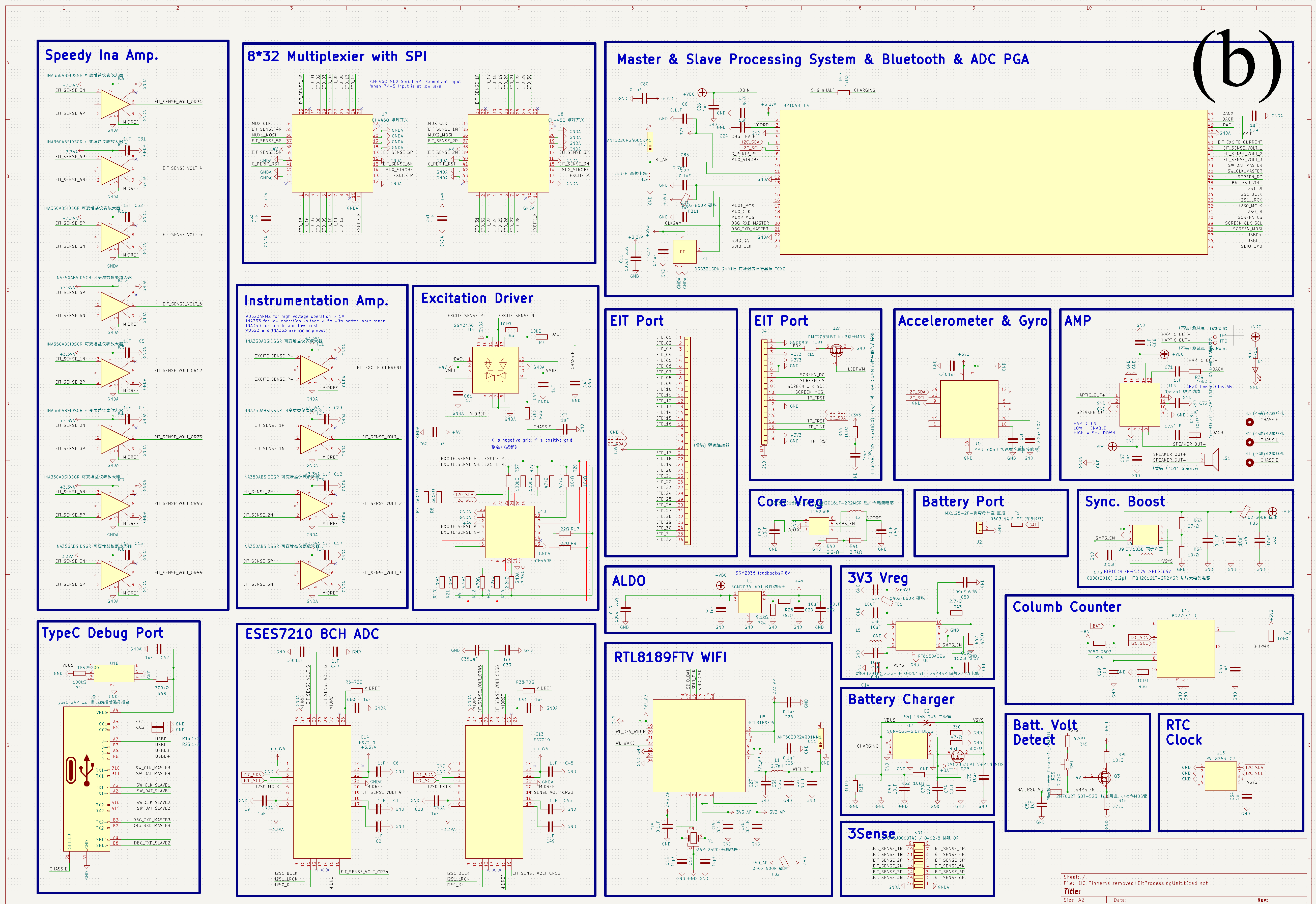


**Figure S3.** a) The second version of the schematic diagram (the Wireless and real-time portable EIT device). b) The third version of the schematic diagram.

**Figure S4.** PCB diagram of the EIT device. a) the first version, b) the second version, and c) the third version.


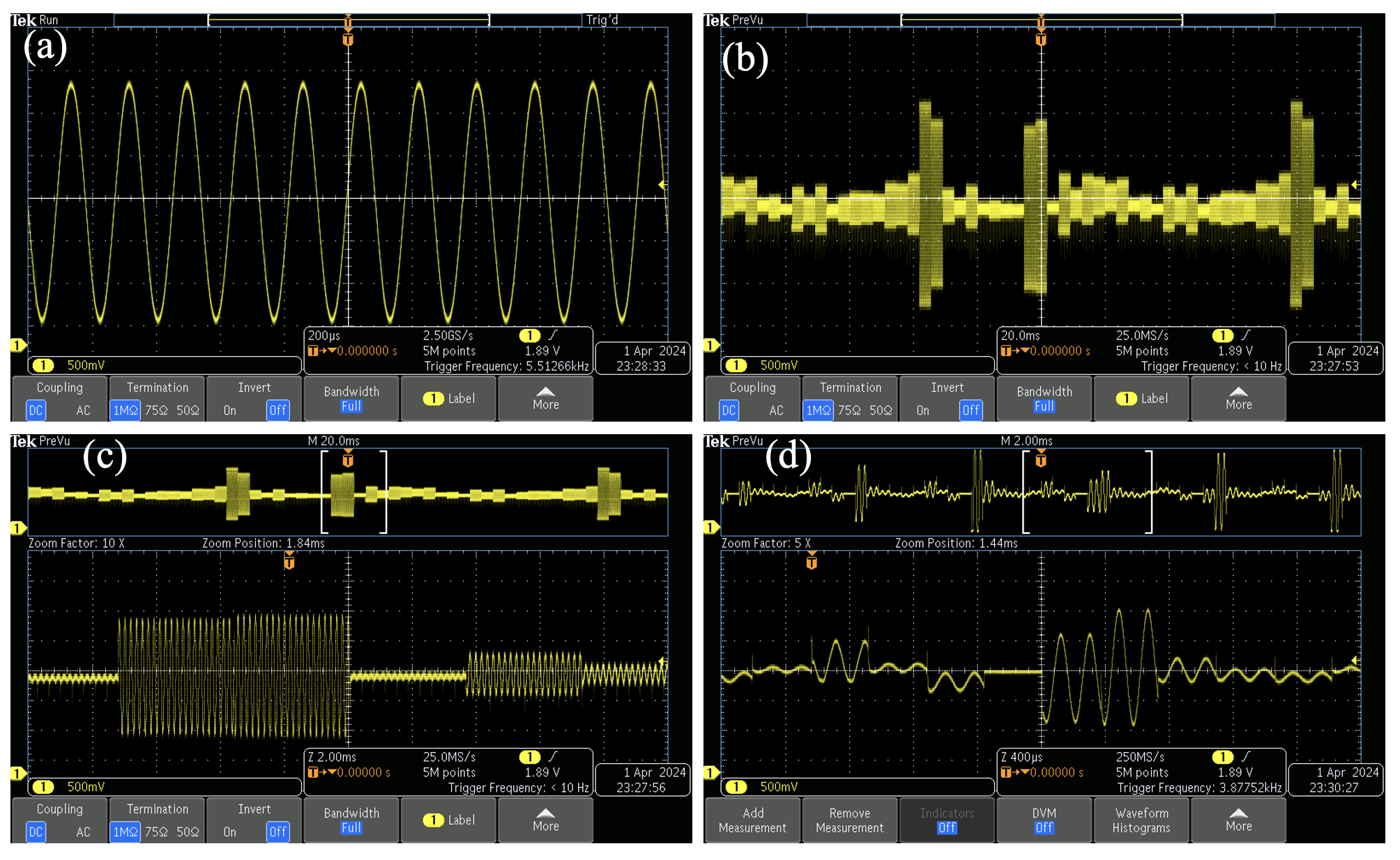


**Figure S5.** a) Excitation voltage of EIT-V3 device. b) Voltage waveform measured on one EIT electrode of the EIT-V3 device. c) Waveform details measured on one EIT electrode of 6 excitation cycles. d) The sampled waveform on the instrumentation amplifier for voltage acquisition.


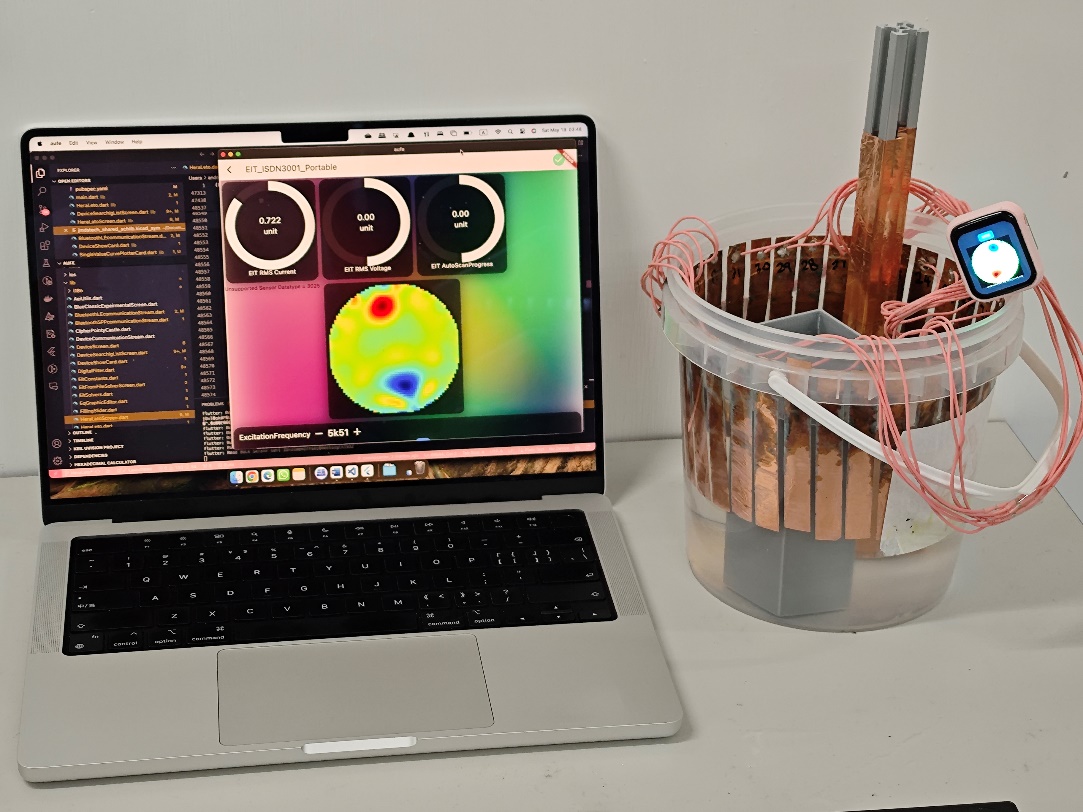


**Figure S6.** Wireless and real-time portable EIT testing setup with a high-conductivity item (Copper foil wrapped on an aluminum stick) and a low-conductivity item put into the testing beaker with copper electrodes array.


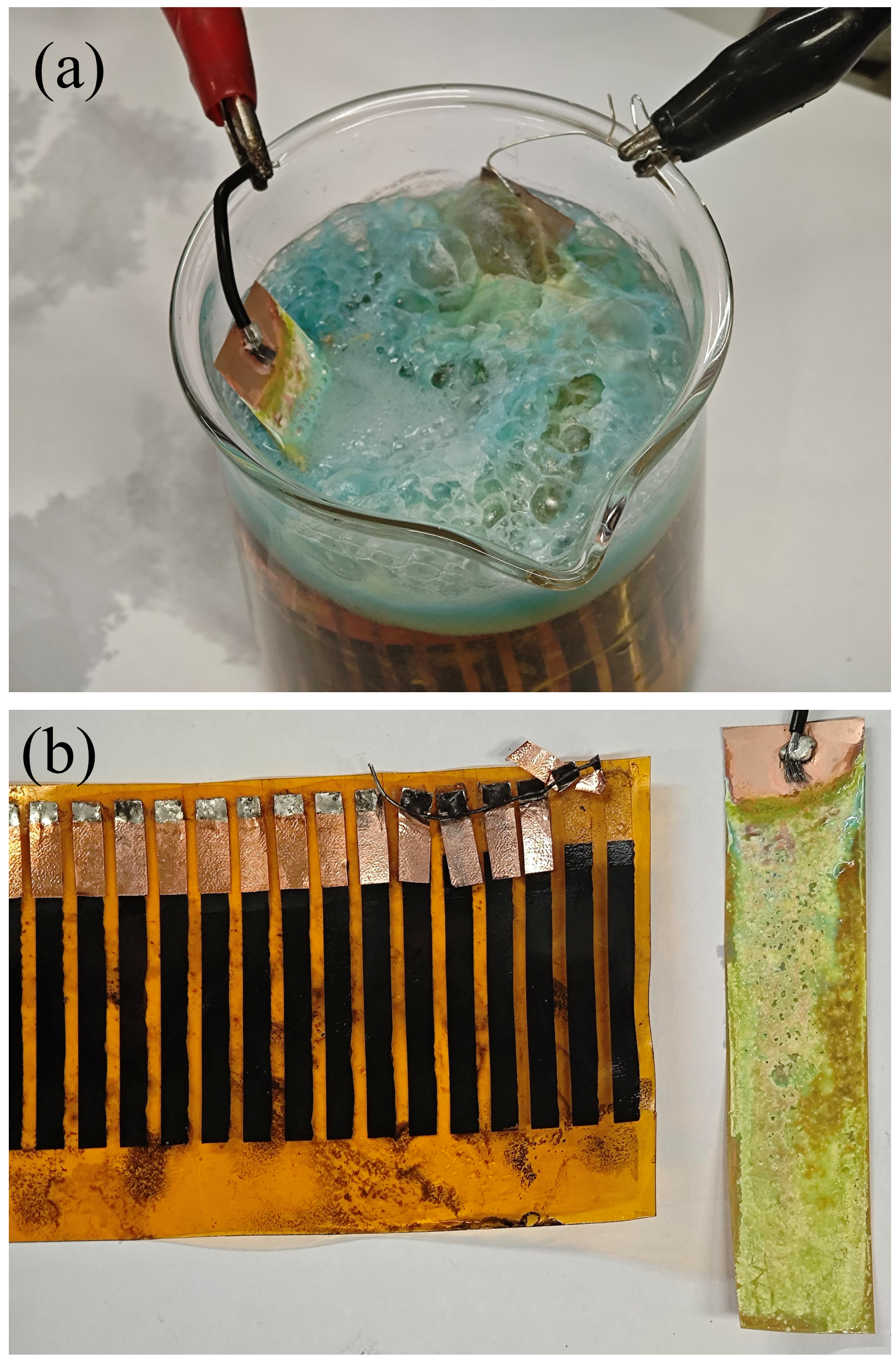


**Figure S7.** Accelerated corrosion comparison experiment with copper and LIG electrodes in seawater. a) Corrosion experiment in progress. b) Comparison between LIG and Copper electrode after a 30-minute corrosion experiment in seawater with 3-volt electrical stimulation.


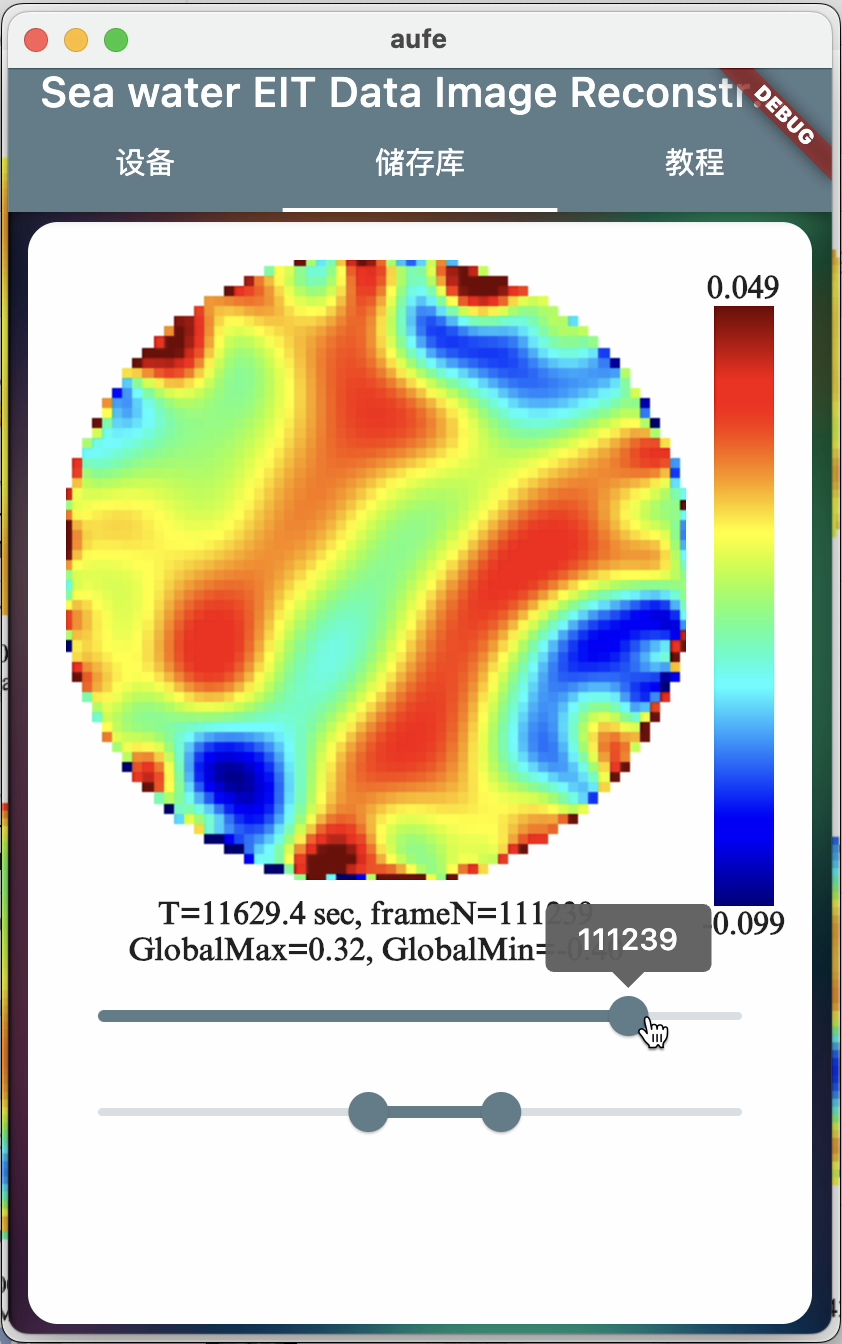


**Figure S8.** Software for solving the EIT inverse problem with the Tikhonov algorithm built via Flutter.
